# Measurement of Klebsiella Intestinal Colonization Density To Assess Infection Risk

**DOI:** 10.1128/mSphere.00500-21

**Published:** 2021-06-23

**Authors:** Yuang Sun, Alieysa Patel, John SantaLucia, Emily Roberts, Lili Zhao, Keith Kaye, Krishna Rao, Michael A. Bachman

**Affiliations:** a Department of Pathology, University of Michigan Medical School, Michigan Medicine, Ann Arbor, Michigan, USA; b DNA Software, Inc., Plymouth, Michigan, USA; c Department of Biostatistics, School of Public Health, University of Michigan, Ann Arbor, Michigan, USA; d Division of Infectious Diseases, Department of Internal Medicine, University of Michigan Medical School, Michigan Medicine, Ann Arbor, Michigan, USA; e Department of Microbiology and Immunology, University of Michigan Medical School, Michigan Medicine, Ann Arbor, Michigan, USA; University of Kentucky

**Keywords:** *Klebsiella*, dominance, infection risk, intestinal colonization, microbiome, qPCR

## Abstract

Klebsiella pneumoniae and the closely related species K. variicola and K. quasipneumoniae are common causes of health care-associated infections, and patients frequently become infected with their intestinal colonizing strain. To assess the association between Klebsiella colonization density and subsequent infections, a case-control study was performed. A multiplex quantitative PCR (qPCR) assay was developed and validated to quantify Klebsiella (K. pneumoniae, *K. variicola*, and *K. quasipneumoniae* combined) relative to total bacterial DNA copies in rectal swabs. Cases of Klebsiella infection were identified based on clinical definitions and having a clinical culture isolate and a preceding or coincident colonization isolate with the same *wzi* capsular sequence type. Controls were colonized patients without subsequent infection and were matched 2:1 to cases based on age, sex, and rectal swab collection date. qPCR from rectal swab samples was used to measure the association between the relative abundance of Klebsiella and subsequent infections. The Klebsiella relative abundance by qPCR was highly correlated with 16S sequencing (ρ = 0.79; *P *< 0.001). The median Klebsiella relative abundance was higher in cases (15.7% [interquartile range {IQR}, 0.93 to 52.6%]) (*n* = 83) than in controls (1.01% [IQR, 0.02 to 12.8%]) (*n* = 155) (*P* < 0.0001). Adjusting for multiple clinical covariates using inverse probability of treatment weighting, a Klebsiella relative abundance of >22% was associated with infection overall (odds ratio [OR], 2.87 [95% confidence interval {CI}, 1.64 to 5.03]) (*P* = 0.0003) and with bacteremia in a secondary analysis (OR, 4.137 [95% CI, 1.448 to 11.818]) (*P *= 0.0084). Measurement of colonization density by qPCR could represent a novel approach to identify hospitalized patients at risk for Klebsiella infection.

**IMPORTANCE** Colonization by bacterial pathogens often precedes infection and offers a window of opportunity to prevent these infections in the first place. Klebsiella colonization is significantly and reproducibly associated with subsequent infection; however, factors that enhance or mitigate this risk in individual patients are unclear. This study developed an assay to measure the density of Klebsiella colonization, relative to total fecal bacteria, in rectal swabs from hospitalized patients. Applying this assay to 238 colonized patients, a high Klebsiella density, defined as >22% of total bacteria, was significantly associated with subsequent infection. Based on widely available PCR technology, this type of assay could be deployed in clinical laboratories to identify patients at an increased risk of Klebsiella infections. As novel therapeutics are developed to eliminate pathogens from the gut microbiome, a rapid Klebsiella colonization density assay could identify patients who would benefit from this type of infection prevention intervention.

## INTRODUCTION

Klebsiella pneumoniae is a leading cause of health care-associated infections (HAIs) (1). Recent studies have shown that Klebsiella variicola and Klebsiella quasipneumoniae are closely related to, yet distinct species from, K. pneumoniae and cause indistinguishable infections (2, 3). These species are part of the K. pneumoniae complex that together pose a serious public health threat.

Klebsiella commonly colonizes hospitalized patients and can cause bacteremia, pneumonia, and urinary tract infections (UTIs). Previous studies show that Klebsiella colonization is significantly associated with subsequent infections, and 80% of infections in colonized patients are caused by an intestinal colonizing strain (4, 5). Increased colonization density may increase the risk of subsequent infection. For example, intestinal domination (defined as at least a 30% relative colonization density) of *Proteobacteria* was associated with subsequent Gram-negative bacteremia in patients undergoing allogeneic hematopoietic stem cell transplantation, and relative and absolute abundances of *Enterobacterales* associate interactively with infection in intensive care patients (6, 7). In long-term acute-care patients, a relative abundance of carbapenem-resistant K. pneumoniae above 22% was a risk factor for bacteremia (8). Similarly, increased relative abundances of Escherichia and *Enterococcus* in the gut are risk factors for corresponding bacteriuria or UTI in kidney transplant patients (9). These studies indicate that in many cases, colonization is a necessary intermediate step before infection.

Understanding the association between Klebsiella colonization and subsequent infections could provide opportunities for the identification of high-risk patients, intervention, and, ultimately, prevention of infection. Additionally, little is known about the association between Klebsiella gut colonization density and specific infection types such as bacteremia, pneumonia, and UTI. Measuring Klebsiella gut density and assessing gut density as a risk factor for various infections may also shed light on the mechanisms of dissemination from the colonized gut to various infection sites. However, the lack of a rapid and reliable assay to quantify Klebsiella relative abundance in the gut has been a hindrance to both research and potential clinical implementation. Here, we report a quantitative PCR (qPCR)-based assay that can quickly and accurately quantify Klebsiella from rectal swab specimens. We employed this assay in a case-control study of colonized patients to assess Klebsiella rectal relative abundance as a risk factor for bacteremia, pneumonia, or UTI and found a significant association after adjusting for clinical variables.

## RESULTS

### *In silico* analysis.

To design a qPCR assay for measuring the relative abundance of the K. pneumoniae complex, 31 K. pneumoniae, K. quasipneumoniae, and K. variicola strains with complete genomes were selected as “inclusivity” for *in silico* analysis (see Data Set S1 in the supplemental material). Additionally, 8 Klebsiella oxytoca and *Raoultella* strains were selected to represent “near-neighbor exclusivity,” and the human genome and common members of the gut microbiome were used as background sequences that should not be detected by the assay. PanelPlex *in silico* analysis was performed, and the *fiu* (also known as *ybiL*) gene was identified as an optimal target for the assay (Table 1). An overall performance score, based on primer and probe thermodynamic stabilities with their targets, as well as any off-target bindings, was computed for each of the 7 assay designs. The *fiu* assay design 1 (overall score of 99.9%) has a predicted probe binding score of 99.5% with all 31 strains in the “inclusivity” set. Regarding the primers, all 31 predicted binding scores of the forward primer (here, The *fiu*-F) are above 50.0%, with 29 above 86.0%. Twenty-four predicted binding scores of the reverse primer (here, *fiu*-R) are above 95.0%, while 6 are close to 50.0%, and 1 is below 50.0%. Additionally, *fiu* assay design 1 was predicted to have no amplifications with any background genomes. Although its primer bindings have variations, its probe binding scores are uniformly excellent. Therefore, *fiu* assay design 1 (here, *fiu* assay) was chosen for further validation. *fiu* is predicted to encode a catecholate siderophore receptor and in K. pneumoniae is upregulated during growth under iron-limited conditions (10). We are not aware of any effects of the polymorphisms that determine primer inclusivity on the pathogenicity of Klebsiella species. To assess the relative abundance of the K. pneumoniae complex, the *fiu* assay and a previously described panbacterial qPCR assay targeting the 23S rRNA gene (11) were combined to construct a multiplex qPCR assay (here, Kp qPCR assay). Overall, the *fiu* assay has good coverage of the K. pneumoniae complex, and the Kp qPCR assay provides a possible solution to quantify the K. pneumoniae complex in clinical specimens.

**TABLE 1:** Primers and probes used in the study

Oligonucleotide	Final concn (nM)	Sequence (5′–3′)^*a*^
*fiu*_Probe	200	FAM-CGTCCACAGCGTAAAGGCATGTT-MGB
23S_Probe	200	VIC-CCTAAGGTAGCGAAATTCCTTGT-MGB
*fiu*-F	400	AACGTAGCGCAGGATGGATCTTCCG
*fiu*-R	400	GACAGATCGCTGGTGGCCTGATA
23S-F	400	ATTACGCCATTCGTGCAGGTCGGA
23S-R	400	TAAACGGCGGCCGTAACTATAACGGT

a FAM, 6-carboxyfluorescein.

10.1128/mSphere.00500-21.4 DATA SET S1 Design parameters of the *fiu* assay. Download Data Set S1, XLSX file, 0.1 MB. Copyright © 2021 Sun et al. 2021 Sun et al. https://creativecommons.org/licenses/by/4.0/ This content is distributed under the terms of the Creative Commons Attribution 4.0 International license.

### K. pneumoniae complex diversity panel.

**FIG 1:**
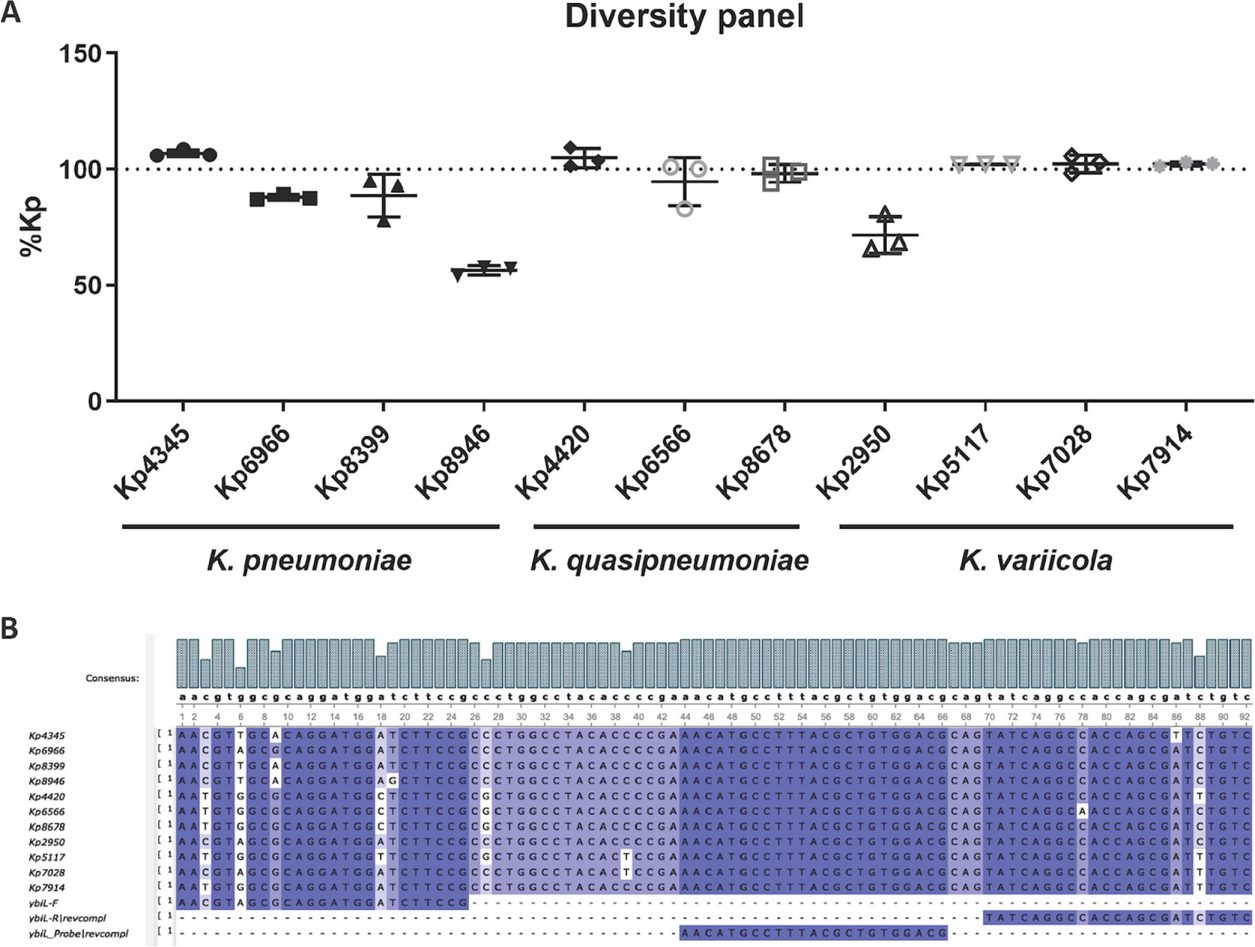
The Kp qPCR assay accurately quantifies K. pneumoniae, *K. variicola*, and *K. quasipneumoniae*. Eleven Klebsiella clinical isolates were extracted and amplified by the Kp qPCR assay, each with 3 technical replicates. (A) Quantification of each isolate relative to KPPR1 set as 100%. (B) Alignment of the amplicons of the 11 isolates with the *fiu* (*ybiL*) primers and probe.

Eleven isolates with polymorphisms at sites of *fiu* primer and probe binding were picked and grown overnight in LB broth. They were resuspended in Amies medium (BD ESwab) and normalized based on CFU for DNA extraction. The laboratory strain ATCC 43816 KPPR1, which contains a single mismatch identical to Kp8399, was set as the reference, and the delta-delta threshold cycle (ΔΔ*C_T_*) method was used to calculate the abundance of Klebsiella relative to KPPR1 (set as 100%) by qPCR (Fig. 1). Of the 11 isolates, 9 are within the range of 88 to >99% relative to KPPR1. Although they share the same polymorphism, the abundance calculation of Kp2950 was 72% relative to KPPR1, whereas that of Kp6966 was 88%. This suggests that technical imprecision may be greater than systematic errors caused by polymorphisms. Taken together, the Kp qPCR assay should have accurate and consistent performance with most clinical isolates despite the existence of binding variations.

### Specificity.

The Kp qPCR assay was designed to quantify K. pneumoniae, *K. quasipneumoniae*, and *K. variicola* but not other Klebsiella species or other common bacteria in the gut microbiota. To validate its specificity, Klebsiella aerogenes, K. pneumoniae subsp. *ozaenae*, K. oxytoca, Raoultella planticola, Raoultella ornithinolytica, Escherichia coli, and Pseudomonas aeruginosa were tested by the Kp qPCR assay (Table S1). K. pneumoniae KPPR1 was used as a positive control. Only KPPR1 and K. pneumoniae subsp. *ozaenae* were amplified by both the *fiu* assay and the 23S assay, whereas K. aerogenes, K. oxytoca, Raoultella planticola, Raoultella ornithinolytica, Escherichia coli, and Pseudomonas aeruginosa strains were amplified only by the 23S assay but not by the *fiu* assay, demonstrating that the *fiu* assay specifically amplified the designated targets but not its near neighbors or background sequences.

10.1128/mSphere.00500-21.2 TABLE S1 Specificity of the Kp qPCR assay Table S1, DOCX file, 0.01 MB. Copyright © 2021 Sun et al. 2021 Sun et al. https://creativecommons.org/licenses/by/4.0/ This content is distributed under the terms of the Creative Commons Attribution 4.0 International license.

### Linearity.

**FIG 2:**
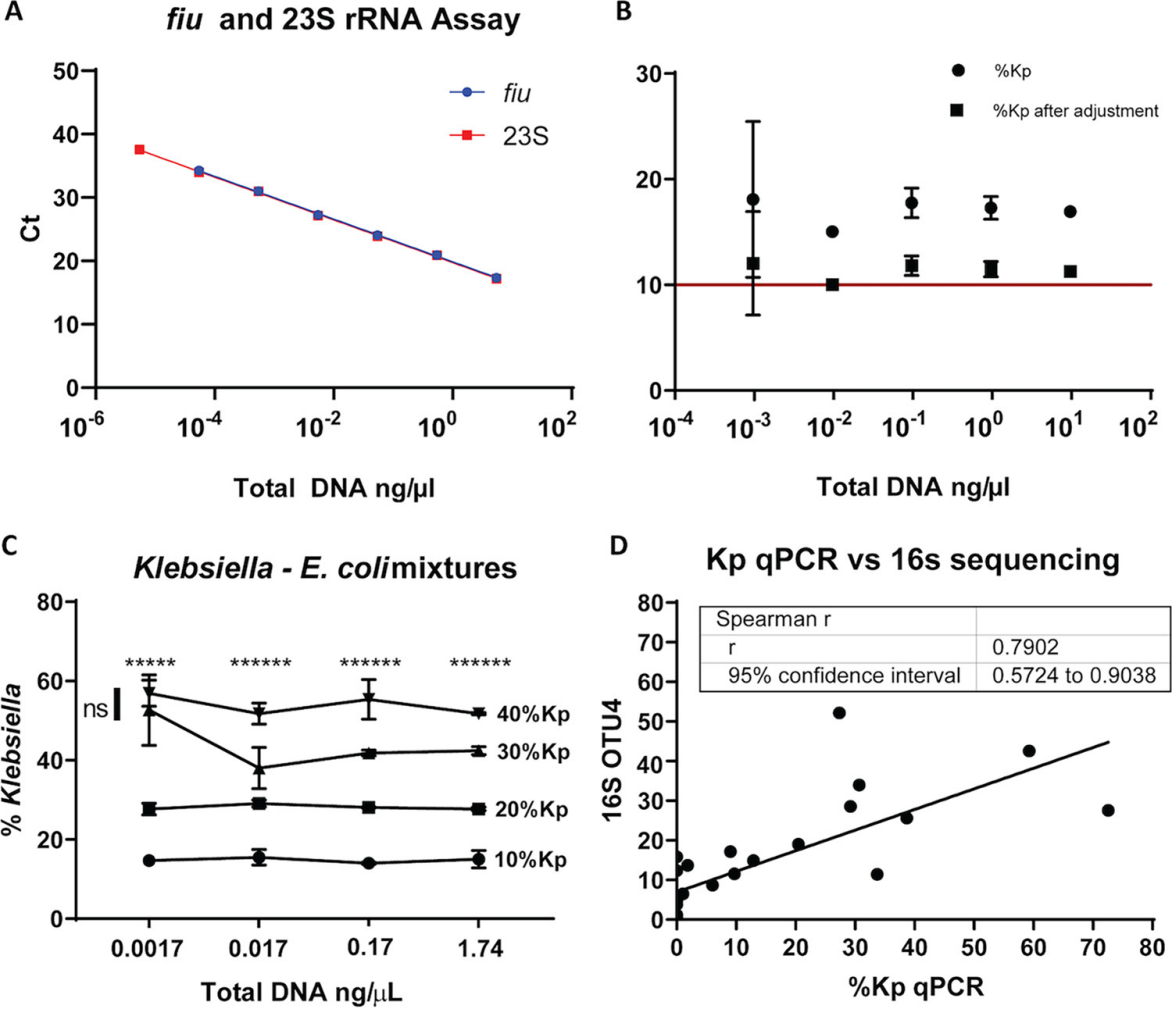
The Kp qPCR assay has the accuracy, precision, and linearity to distinguish small differences in Klebsiella relative abundance. Linearity was assessed with serial dilutions of KPPR1 genomic DNA (*n* = 3 technical replicates) (A). Precision and accuracy were assessed with serial dilutions of a mixture of 89% Bacteroides ovatus, 10% K. pneumoniae KPPR1, and 1% Serratia marcescens genomic DNAs (3 technical replicates) (B). Means and SD of both direct and adjusted quantifications, after consideration of 23S gene copy number variations are shown. Ability to discern relative differences using serial dilutions of mixtures of KPPR1 and Escherichia coli CFT073 (3 technical replicates) (C). For each dilution, one-way ANOVA (*P *< 0.0001 for all) and Tukey’s posttest (* for each comparison out of six with *P* values of <0.05) were performed. ns, not significant. Accuracy was compared to that of 16S rRNA sequencing using OTU4 that contains Klebsiella (D). The correlation between Klebsiella relative abundances by Kp qPCR and OTU4 of 16S rRNA sequencing analysis that contains Klebsiella was measured by Spearman’s rank correlation coefficient on 26 rectal swab samples.

To assess the Kp qPCR assay’s linearity, KPPR1 was grown in LB broth overnight and resuspended in Amies medium. A serial 10-fold dilution was made in triplicate and enumerated for CFU counts. The CFU counts were close to a theoretical 10-fold dilution, as the slope was 0.9889 and the *R*^2^ value was 0.9999. The slopes of the *fiu* and 23S assays were both 3.37, and the *R*^2^ values were both 0.9994, demonstrating that both assays had good linearity and efficiency (Fig. 2A).

### Precision and accuracy.

To assess the precision and accuracy of the Kp qPCR assay, a mixture of 89% Bacteroides ovatus, 10% KPPR1, and 1% Serratia marcescens by DNA quantifications was made and diluted 10-fold serially. The mixture was amplified by the Kp qPCR assay, and the relative abundances of Klebsiella were calculated relative to KPPR1 using the ΔΔ*C_T_* method (Fig. 2B). The relative abundances of Klebsiella ranged from 15.0 to 18.1%, with a mean value of 17.0% (expect 10.0%). The standard deviations (SD) ranged from 0.246 to 7.386. The quantifications by the Kp qPCR assay were consistent across concentrations of 4-log_10_ differences. When the total DNA concentrations of the mixture were over 1 × 10^−2^ ng/μl, the SD of the relative abundances of Klebsiella were less than 1.5%. However, at a total DNA concentration of 1 × 10^−3^ ng/μl, the assay became less precise, as the SD increased to 7.39%. At a total DNA concentration of 1 × 10^−4^ ng/μl, the *fiu* assay did not detect Klebsiella. The copy number of the 23S rRNA gene is organism specific, with 5, 7, and 8 copies in Bacteroides ovatus, Serratia marcescens, and K. pneumoniae, respectively. After adjustment for these differences, the calculated relative abundance of Klebsiella ranged from 10.0 to 12.0%, with a mean value of 11.3% (SD, 0.163 to 4.91%), demonstrating that the Kp qPCR assay can accurately quantify Klebsiella from contrived samples. Fortunately, a bias in the calculation based on the 23S copy number in the overall population relative to Klebsiella would not be expected to impact the ability to measure relative differences, as demonstrated below.

### Accuracy: relative differences.

To assess the Kp qPCR assay’s ability to distinguish different relative abundances of Klebsiella, KPPR1 and Escherichia coli O6:K2:H1 CFT073 were mixed in Amies medium according to CFU counts to make 10%, 20%, 30%, and 40% Klebsiella mixtures. Tenfold serial dilutions were made from each mixture, and genomic DNAs of each serial dilution were isolated and then amplified by the Kp qPCR assay. The relative abundance of Klebsiella was calculated relative to KPPR1 using the ΔΔ*C_T_* method (Fig. 2C). At total DNA concentrations from ∼0.02 to 2 ng/μl, the assay was able to detect relative differences between all dilutions, and at 0.002 ng/μl, it can tell all differences except between 30 and 40% Klebsiella. At all but the lowest total bacterial concentration, the assay can reliably detect 10% differences in Klebsiella relative abundances.

### Accuracy in comparison to 16S rRNA sequencing.

To compare the relative abundance calculated by qPCR to that calculated by the gold standard of 16S rRNA sequencing, 26 residual samples with 16S rRNA sequence data from a previous study were analyzed (Fig. 2D) (11). Klebsiella relative abundances by Kp qPCR were highly correlated with operational taxonomic unit 4 (OTU4) that contained reads identical to Klebsiella 16S rRNA sequences (Spearman’s ρ = 0.79; *P* < 0.001).

### Case-control study.

To assess the association between Klebsiella colonization density and subsequent infection, a nested case-control study of colonized patients was performed at a single large academic medical center in Michigan. We sequentially enrolled 1,978 subjects from 2,087 separate admissions with Klebsiella colonization in a rectal swab, collected as part of routine surveillance for vancomycin-resistant *Enterococcus* in intensive care and oncology wards. Of these colonized subjects, 83 cases were identified that met clinical definitions of subsequent infection and had an infecting isolate that matched a colonizing isolate on or prior to the day of infection by *wzi* sequence typing. This comprised 41 bloodstream infections, 19 respiratory infections, and 23 urinary tract infections with concordant infecting and colonizing isolates. Controls were defined as colonized patients who had no documented Klebsiella infection but had a negative clinical culture collected of the same type as that of the matching case. Each case was matched to two controls based on age, sex, date of rectal swab collection, and swab availability, for a total of 155 controls. To find matches for every case, the criteria for age were modified for 2 cases (±20 years), and the criteria for swab collection date were modified for 4 cases (±118 days). Cases had a significantly higher comorbidity score and were more likely to have exposure to diuretics, vitamin D, and a vasopressin blocker prior to the rectal swab. They were also more likely to have been exposed to high-risk antibiotics associated with disruption of the intestinal microbiome (12). Cases also had lower baseline albumin levels and were more likely to have a urinary catheter or feeding tube prior to the rectal swab. There was no significant association between intensive care unit (ICU) or hematology/oncology ward location and case status (Table 2). The median numbers of days (IQR) from swab collection to infection were 10.00 (25.00) days overall, 3.50 (14.75) days for bloodstream infections, 23.50 (44.00) days for urinary tract infections, 10.00 (14.50) days for pneumonia, and 3.50 (8.00) days for other sites.

**TABLE 2:** Patient demographics

Variable^*a*^			*P* value (logistic regression)
	Cases (*n* = 83)	Controls (*n* = 155)	
Mean age (yrs) ± SD	60.08 ± 12.90	59.43 ± 12.29	0.759
Male patients	44 (53.0)	83 (53.5)	>0.99
White patients	70 (84.3)	122 (78.7)	0.368
Mean Elixhauser comorbidity score ± SD	7.53 ± 3.25	6.62 ± 3.12	0.05
Mean weighted Elixhauser score ± SD	22.40 ± 11.53	19.36 ± 11.89	0.104
Depression	29 (34.9)	40 (25.8)	0.203
Diuretic	30 (36.1)	36 (23.2)	0.03
Vitamin D	18 (21.7)	18 (11.6)	0.032
Vasopressin blocker	19 (22.9)	15 (9.7)	0.008
Broad-spectrum antibiotic^*b*^	30 (36.1)	30 (19.4)	0.005
Mean baseline albumin level (g/dl) ± SD	2.53 ± 0.71	2.78 ± 0.73	0.008
Albumin level of ≥2.5 g/dl	46 (55.4)	112 (72.3)	0.003
Urinary catheter	60 (72.3)	86 (55.5)	0.016
Feeding tube	7 (8.43)	3 (1.94)	0.023
Ventilator	35 (42.2)	66 (42.6)	>0.99
Central line	34 (41.0)	74 (47.7)	0.301
Ward			
ICU	59 (71.1)	99 (63.9)	0.256
Oncology	24 (28.9)	56 (36.1)	

a All variables are baseline features at the time of swab collection. The values given are no. (%) per group, unless otherwise specified.

b Defined as exposure to any of the following in the 90 days prior to Klebsiella colonization: third- or fourth-generation cephalosporins, fluoroquinolones, lincosamides, β-lactam/β-lactamase inhibitor combinations, oral vancomycin, and carbapenems (12).

**FIG 3:**
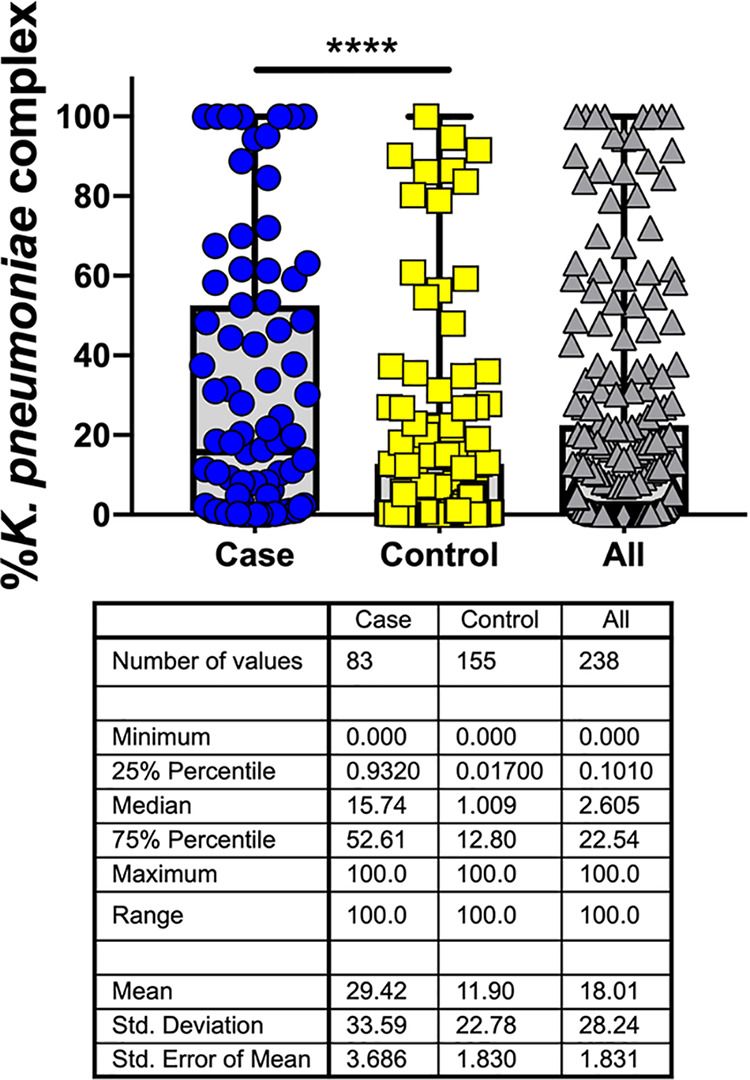
Increased relative abundance of Klebsiella is associated with subsequent infection. The relative abundance of Klebsiella in rectal swabs as measured by the Kp qPCR assay is shown for 238 specimens, with 83 cases matched 1:2 to 155 controls based on age, sex, and date of swab collection. Medians and interquartile ranges are shown. ****, *P* < 0.0001 by unadjusted conditional logistic regression.

To assess colonization density, the Kp qPCR assay was performed on all of the rectal swab samples (Fig. 3). The overall DNA concentration in the rectal swabs fell within the validated performance range of the assay (median, 0.67 ng/μl; interquartile range, 0.18 to 1.83 ng/μl; range, 0 to 13.44 ng/μl). The median relative abundance was 2.61% overall, with an IQR of 0.1 to 22.5% and an overall range of 0 to 100%. In cases, the median was 15.74% (IQR, 0.93 to 52.61%), and in controls, the median was 1.01% (IQR, 0.02 to 12.8%). To determine if dominance was associated with infection while accounting for case-control matching, a cutoff for dominance was applied. The 75th percentile of 22% in the overall data set was chosen, consistent with cutoffs of 22% and 30% used in previous studies (6, 8). Subjects with a K. pneumoniae gut colonization density of >22% had a 3.34-fold (range, 1.95- to 5.72-fold) (*P* < 0.0001) increased odds of infection compared to those with lower colonization density levels.

To adjust for patient variables associated with Klebsiella infection, inverse probability of treatment weighting (IPTW) was used. In the overall cohort of 1,978 subjects, an explanatory model for invasive infection was built using baseline clinical features at the time of colonization (13) (Table S2). The model built by purposeful selection selected the following variables for inclusion: Elixhauser score, depression, diuretic use, vitamin D use, use of pressors/inotropes, low serum albumin levels (<2.5 g/dl), and exposure to antibiotics with a high risk of microbiome disruption (Table S1). These variables were then used to model a K. pneumoniae gut colonization density of >22% to generate weights for the nested case-control study. Using weights derived from these clinical covariates (available in 228 of 240 subjects), patients with a K. pneumoniae gut colonization density of >22% had a 2.87-fold (range, 1.64- to 5.03-fold) (*P* = 0.0003) increased odds of infection compared to those with lower colonization density levels. ICU status was added as a separate variable in the model and was not associated with case status (*P *= 0.243). In a secondary analysis restricted to infections within 90 days of infection (*n* = 75), the association between a colonization density of >22% and infection was also significant (odds ratio [OR], 3.014 [95% confidence interval {CI}, 1.725 to 5.267]) (*P *= 0.0001). In a secondary analysis by site of infection, an increased relative abundance was also significantly associated with bloodstream infection (OR, 4.137 [95% CI, 1.448 to 11.818]) (*P *= 0.0084), whereas associations with urinary tract (OR, 3.037 [95% CI, 0.571 to 16.17]) (*P* = 0.19) and respiratory (OR, 1.32 [95% CI, 0.38 to 4.565]) (*P* = 0.66) infections did not reach significance.

10.1128/mSphere.00500-21.3 TABLE S2 Clinical explanatory model of invasive Klebsiella infection following colonization in the overall cohort (1,978 subjects from 2,087 admissions). Download Table S2, DOCX file, 0.01 MB. Copyright © 2021 Sun et al. 2021 Sun et al. https://creativecommons.org/licenses/by/4.0/ This content is distributed under the terms of the Creative Commons Attribution 4.0 International license.

## DISCUSSION

The goal of this study was to measure the association between Klebsiella colonization density and subsequent infection. To develop a robust method that accurately and precisely measured the relative abundances of K. pneumoniae, *K. quasipneumoniae*, and *K. variicola* among the gut microbiota, we developed a novel qPCR assay for detecting these dominant members of the K. pneumoniae complex and combined it with measurement of 23S rRNA gene copies. Analytical validation indicated that this assay is inclusive of multiple strains of each species and is able to distinguish as little as 10% differences in relative abundance between samples. Applying this assay to a case-control study of Klebsiella infections among colonized, intensive care patients indicated that increased Klebsiella density is associated with subsequent infection in both unadjusted and adjusted analyses.

The finding that Klebsiella colonization density is associated with subsequent infection raises several interesting possibilities. One is that infection risk is dictated by how much Klebsiella is present in the gut, independent of the varying gene content of Klebsiella strains. Indeed, we and others have shown that detectable colonization is associated with infection (4, 5), and the lower limit of detection for culture is an indirect measure of abundance in rectal swabs. However, we have also demonstrated that particular Klebsiella genes are associated with infection as opposed to asymptomatic colonization (14), indicating that which strain a patient is colonized with affects their risk. There is likely to be an important interaction between Klebsiella gene content and colonization density, where certain genes may increase gut fitness and, therefore, gut abundance. Alternatively, there may be strains where gut abundance is increased based on microbiome factors extrinsic to Klebsiella but where the risk of infection is further increased by virulence genes that act at the site of infection. Finally, Klebsiella strains with fitness genes that increase abundance in the gut and virulence genes that act later in pathogenesis are likely to pose the greatest risk of infection in colonized patients. In future studies, comparative genomics could be performed to identify genes associated with infection while simultaneously measuring the relative abundance of these strains on patient rectal swabs. Genes associated with infection could also be evaluated for their association with intestinal dominance and characterized for their function during colonization. Genes that increase the risk of infection through intestinal dominance will be of particular interest in devising approaches to clear Klebsiella colonization.

The main limitation of this study was the relatively small number of cases (*n* = 83). We compensated for this by using a case-control design and an inverse probability of treatment weighting to account for clinical variables potentially associated with infection and intestinal dominance without a significant loss of statistical power (15). However, we were limited in our ability to investigate associations by site of infection. Bloodstream infections were the largest infection type and were independently associated with intestinal dominance. Intriguingly, the point estimate for urinary tract infections (3.037) was similar to the overall odds ratio (2.87) for infection. This may indicate that intestinal dominance is also associated with UTIs, perhaps because a key step in pathogenesis is thought to be the transit of intestinal bacteria across the perineum to the urethra. The case-control study design itself could be considered a limitation. However, given the rarity of our primary outcome, a cohort design would have been inefficient and underpowered. A downside of case-control designs is the risk of bias and confounding. Since our study population was nested within a cohort and our controls were randomly chosen (when multiple matches were possible for each case), bias risk was mitigated. The risk of unmeasured confounding was further mitigated since we were able to use our companion cohort study (13), where sufficient power was present, to fully explore and identify the salient clinical confounders needed for adjustment. Thus, since we used a cohort study first to identify clinical covariates and already included all of the cases, the only additional benefit of adding more controls would come from increased precision in our estimate of Klebsiella density among controls. However, our estimate of density in controls is already fairly precise (11.9% [standard error of the mean {SEM}, 1.83%; CI, 8.28 to 15.51%]) and is separated greatly from cases, even comparing the confidence intervals (29.42% [SEM, 3.69%; CI, 22.09 to 36.75%]), so adding many more controls at a considerable expense is unlikely to have changed our central conclusion.

This study further supports the growing paradigm that intestinal dominance can be used to predict infections in our hospitals. Previous studies have demonstrated that the dominance of carbapenemase-producing K. pneumoniae in long-term-care patients (8) and K. pneumoniae in allogeneic stem cell transplant patients (16) is associated with infection. This study evaluated a more heterogeneous population of intensive care patients with a combined outcome of bloodstream, respiratory, or urinary tract infections and found the same association. The successful use of qPCR demonstrates the feasibility of measuring the relative abundance of targeted pathogens in the gut using methods that are standard in clinical microbiology laboratories and inexpensive relative to next-generation sequencing. This a key step in moving toward infection prevention in hospitalized patients. qPCR could be applied to detect colonization in rectal swabs as well as quantify it in a single step, thereby incorporating two levels of Klebsiella infection risk. Combined with the assessment of patient risk factors and perhaps targeted testing for Klebsiella virulence genes, an integrated risk assessment could be performed. If this relative risk is high enough, infection prevention interventions should be considered. Fortunately, safe and effective therapeutic strategies to eliminate gut colonization by pathogens are emerging, and results from fecal transplant studies are encouraging (17). In the near future, it may be possible to assess the risk of a carbapenem-resistant Klebsiella infection at the time of hospital admission and prevent it without the use of antibiotics.

## MATERIALS AND METHODS

### Study design and subject enrollment.

To assess the role of Klebsiella colonization density in the risk of subsequent invasive infection, we conducted a nested case-control study drawn from a larger cohort of 1,978 patients consecutively enrolled from 2,087 inpatient admissions. Subjects admitted to intensive care units (ICUs) and oncology wards at our hospital undergo routine surveillance by rectal swab culture for vancomycin-resistant *Enterococcus*. After such testing, we collected residual media from these swabs and enrolled subjects in our study if colonization with K. pneumoniae or *K. variicola* was detected by selective culture on MacConkey agar and confirmed by matrix-assisted laser desorption ionization–time of flight (MALDI-TOF) identification (Bruker MALDI Biotyper). Cases were identified from this larger cohort and matched to controls as described below.

### Case definitions.

Michigan Medicine patients from ICUs and select wards (hematology, oncology, and hematopoietic stem cell transplant) with Klebsiella colonization based on a rectal swab culture and a positive Klebsiella blood, respiratory, or urine culture were identified as putative cases. Manual chart review was conducted by the study team to decide if the patients met clinical definitions of pneumonia or urinary tract infections (18–22). All patients with a Klebsiella blood culture were considered to have an infection. For those meeting clinical case definitions of infection, the clinical isolate and preceding rectal swab isolates were evaluated for concordance by *wzi* gene sequencing as previously described (4, 23). Although not as powerful as core-genome multilocus sequence typing (MLST), we have previously demonstrated that *wzi* sequencing has a discriminatory power similar to that of 7-gene multilocus sequence typing (4). Patients with concordant infection and colonizing isolates were considered cases. Controls were defined as colonized patients who had no documented Klebsiella infection but had a negative culture collected of the same type as that of the matching case. Cases and controls were matched 1:2 based on age ± 10 years, sex, and rectal swab collection date ± 90 days. This study was approved by the University of Michigan Institutional Review Board.

### Samples for PCR analysis.

Rectal swabs were collected using the ESwab collection and transport system (Becton, Dickinson, Franklin Lakes, NJ, USA), which elutes the sample into 1 ml Amies medium. Unless specified otherwise, contrived samples that were used in PCR analysis were eluted in the Amies medium as well. The 89% Bacteroides ovatus, 10% KPPR1, and 1% Serratia marcescens mixtures were suspended in double-distilled water (ddH_2_O).

### Bacterial DNA extraction.

Genomic DNA was isolated using the MagAttract PowerMicrobiome DNA/RNA kit (Qiagen, Germantown, MD, USA) on the epMotion 5075 liquid handler (Eppendorf, Hauppauge, NY, USA). A volume of 100 μl was added to the bead plate for each rectal swab and contrived sample. Subsequent steps of DNA extraction were conducted according to the manufacturer’s instructions. Bacteroides ovatus, KPPR1, and Serratia marcescens cultures were extracted using DNeasy blood and tissue kits (Qiagen, Germantown, MD, USA) according to the manufacturer’s instructions for Gram-negative bacteria. Residual DNA eluates from rectal swab samples previously characterized by 16S sequencing were also used in this study.

### *In silico* assay design.

PanelPlex (DNA Software, Ann Arbor, MI, USA) *in silico* analysis was performed. Thirty-one K. pneumoniae, *K. quasipneumoniae*, and *K. variicola* genomes were selected as “inclusivity”; 8 K. oxytoca and *Raoultella* strains were selected as “near-neighbor exclusivity”; and the human genome and common members of the gut microbiome were selected as background sequences that should not be detected by the assay (see Data Set S1 in the supplemental material). PanelPlex utilizes ThermoBLAST technology to scan for thermodynamically stable off-target hybridizations that cause false-positive test results.

### Quantitative PCR assay.

Real-time PCR was performed using primers (Integrated DNA Technologies, Coralville, IA, USA) and probes (Thermo Fisher Scientific, Waltham, MA, USA) with sequences and concentrations listed in Table 1 in combination with the QuantiTect multiplex PCR kit (Qiagen, Germantown, MD, USA). PMAxx (Biotium, Fremont, CA, USA) at a final concentration of 6 μM was added. A volume of 5 μl was used for each template. The final reaction volume was 25 μl. Prior to template addition, the reaction mixture was incubated for 10 min at room temperature and then treated in a Biotium PMA-Lite light-emitting diode (LED) photolysis device for 10 min. PCR conditions were 50°C for 2 min, 95°C for 15 min, and then 40 cycles of 94°C for 1 min and 60°C for 1 min on a QuantStudio 3 real-time thermocycler (Thermo Fisher Scientific, Waltham, MA, USA). KPPR1 genomic DNA was used as a positive control and 100% reference for calculating Klebsiella relative abundances. Relative abundance was calculated using the ΔΔ*C_T_* method, i.e., $\text{relative abundance }=2^{\left(\Delta C_{T}{}_{\text{Sample}}-\Delta C_{T}{}_{\text{KPPR}1}\right)}\times 100\%$, where Δ*C_T_* = *C_T_*_23S_ − *C_TybiL_*. Relative abundance was capped at 100%.

### Statistical analysis.

Linearity was validated by linear regression. Spearman’s rank correlation coefficient was used for correlation between Kp qPCR and 16S rRNA sequencing. One-way analysis of variance (ANOVA) and Tukey’s posttest were performed to compare each dilution of the KPPR1 and Escherichia coli O6:K2:H1 CFT073 mixtures. Statistical analysis was performed by using Prism 8 (GraphPad, San Diego, CA, USA).

### Clinical modeling.

Conditional logistic regression was used to study the effect of the relative abundance of colonization on Klebsiella infection while accounting for case-control matching. Unadjusted analysis was performed after dichotomizing the relative abundance at the third quartile of 22%. To adjust for patient variables associated with Klebsiella infection, an inverse probability of treatment weighting (IPTW) approach was used. Although IPTW is often used to compare treatments, it can be used to estimate the relationship between any nonrandom factor and the outcome of interest (15). In this study, we used IPTW for colonization density >22% in the estimation of its effect on the outcome of infection. Given the smaller sample size in our nested case-control study, we turned to the larger cohort from which our subjects were derived to identify most robustly the clinical variables that best explain the risk of infection (13). First, using the increased power afforded by the overall cohort of 1,978 subjects, an explanatory unconditional logistic regression model for invasive infection was built using baseline clinical features at the time of colonization. The model was built by purposeful selection, a common technique (24). Briefly, purposeful selection begins with an unadjusted analysis of each variable to select candidates with statistically significant associations with the outcome, and these are included in the starting set of covariates for the multivariable model. Iteratively, covariates are then removed from the model if they are nonsignificant (*P *> 0.05) and not a confounder (i.e., they do not affect the estimate of other variables’ coefficients by at least 20%). A change in a parameter estimate above the specified level indicates that the excluded variable was important in the sense of providing a needed adjustment for one or more of the variables remaining in the model (i.e., it should be retained even if not significant). The resulting model contains significant covariates and other confounders, and variables not included are then added back one at a time. Once again, the model is iteratively reduced as described above but only for the variables that were additionally added. At the end of this final step, we are left with a multivariable model for Klebsiella infection drawn from the larger cohort of subjects with rectal Klebsiella colonization. The variables selected for inclusion by this method were then used to generate propensity scores for a Klebsiella colonization density of >22% but only for subjects in the nested case-control study, again via unconditional logistic regression. The propensity scores were then used to generate weights for the IPTW process and subsequent weighted conditional logistic regression for Klebsiella infection. The inverses of these propensity scores were then used as weights in the subsequent weighted conditional logistic regression for Klebsiella infection with robust standard errors. Both unadjusted and adjusted analyses were conducted using the proc survveylogistic procedure in SAS (version 9.4), and covariate balance was assessed using the cobalt package in R.

### Data availability.

16S sequencing samples from rectal swabs PR08714, PR11216, PR05497, PR09929, PR10907, PR05713, PR06107, PR08411, PR08133, PR05629, PR08147, PR07331, PR12066, PR07876, PR08427, PR07976, PR08661, PR05017, PR08962, PR09113, PR08102, PR09612, PR08748, PR08048, PR06316, and PR10214 are available in the Sequence Read Archive under accession number PRJNA641414.

10.1128/mSphere.00500-21.1 FIG S1 Balance plot of IPTW. Download FIG S1, DOCX file, 0.1 MB. Copyright © 2021 Sun et al. 2021 Sun et al. https://creativecommons.org/licenses/by/4.0/ This content is distributed under the terms of the Creative Commons Attribution 4.0 International license.
