# Supplementary material for: Measurement of Klebsiella Intestinal Colonization Density To Assess Infection Risk
Source: mSphere. 2021 Jun 23;6(3):e00500-21. doi: 10.1128/mSphere.00500-21 (PMC8265666; doi:10.1128/mSphere.00500-21)
Supplement: TABLE S1 [file msphere.00500-21-st001.docx]

Table S1. Specificity of the Kp qPCR assay

| Species | Description | C_T_ 23S | C_T_ *fiu* | %Kp (ddC_T_ method) |
| --- | --- | --- | --- | --- |
| *K. pneumoniae* | KPPR1; Positive control | 13.64 | 16.36 | Reference (100%) |
| *K. aerogenes* | Clinical Isolate Kp3025 | 13.33 | > 37 | 0 |
| *K. pneumoniae subsp. Ozaenae* | Clinical Isolate Kp173 | 13.49 | 16.53 | 79.27 |
| *K. oxytoca* | Clinical Isolate Kp7040 | 13.20 | > 37 | 0 |
| *K. oxytoca* | Clinical Isolate Kp7049 | 13.60 | > 37 | 0 |
| *K. oxytoca* | Clinical Isolate Kp7058 | 14.23 | > 37 | 0 |
| *K. oxytoca* | Clinical Isolate Kp7061 | 13.24 | > 37 | 0 |
| *Raoultella planticola* | Clinical Isolate Kp75 | 15.09 | > 37 | 0 |
| *Raoultella ornithinolytica* | Clinical Isolate Kp2777 | 13.794 | > 37 | 0 |
| *Escherichia coli* | CFT073 | 13.46 | > 37 | 0 |
| *Pseudomonas aeruginosa* | PA01 | 13.196788 | > 37 | 0 |
| *Ct of 37 was used as the cutoff for reactivity* | | | | |
