# Supplementary material for: Measurement of Klebsiella Intestinal Colonization Density To Assess Infection Risk
Source: mSphere. 2021 Jun 23;6(3):e00500-21. doi: 10.1128/mSphere.00500-21 (PMC8265666; doi:10.1128/mSphere.00500-21)
Supplement: TABLE S2 [file msphere.00500-21-st002.docx]

Table S2. Clinical explanatory model of invasive *Klebsiella* infection following colonization in the overall cohort (1978 subjects from 2087 admissions).

|  | **OR** | **95% CI** | | ***P*** |
| --- | --- | --- | --- | --- |
| Elixhauser Score (weighted) | 1.02 | 1.00 | 1.04 | .036 |
| Depression | 1.80 | 1.11 | 2.87 | .015 |
| Prior diuretic use | 1.28 | 0.70 | 2.28 | .413 |
| Prior vitamin D use | 1.36 | 0.70 | 2.55 | .349 |
| Prior use of pressors | 1.86 | 0.94 | 3.63 | .071 |
| Prior use of broad-spectrum antibiotics^1^ | 1.41 | 0.75 | 2.56 | .274 |
| Albumin < 2.5 g/dL | 2.11 | 1.29 | 3.44 | .003 |
| ^1^Defined as exposure to any of the following in the 90 days prior to *Klebsiella* colonization: third- or fourth-generation cephalosporins, fluoroquinolones, lincosamides, β-lactam/ β -  lactamase inhibitor combinations, oral vancomycin, and carbapenems | | | | |
