# Supplementary material for: Measurement of Klebsiella Intestinal Colonization Density To Assess Infection Risk
Source: mSphere. 2021 Jun 23;6(3):e00500-21. doi: 10.1128/mSphere.00500-21 (PMC8265666; doi:10.1128/mSphere.00500-21)
Supplement: FIG S1 [file msphere.00500-21-sf001.docx]

Figure S1. Balance plot showing standardized differences of each covariate in the propensity score before and after IPTW weighting. Differences closer to 0 demonstrate better balance in the covariate distribution between those with high and low density.
